# Mammalian Sulfatases: Biochemistry, Disease Manifestation, and Therapy

**DOI:** 10.3390/ijms23158153

**Published:** 2022-07-24

**Authors:** Ryuichi Mashima, Mahito Nakanishi

**Affiliations:** 1 Department of Clinical Laboratory Medicine, National Center for Child Health and Development, 2-10-1 Okura, Setagaya-ku, Tokyo 157-8535, Japan; 2 TOKIWA-Bio Inc., 2-1-6 Sengen, Tsukuba 305-0047, Japan; nakanishi@tokiwa-bio.com

**Keywords:** sulfatase, post-translational modification, formylglycine, biochemistry, gene therapy

## Abstract

Sulfatases are enzymes that catalyze the removal of sulfate from biological substances, an essential process for the homeostasis of the body. They are commonly activated by the unusual amino acid formylglycine, which is formed from cysteine at the catalytic center, mediated by a formylglycine-generating enzyme as a post-translational modification. Sulfatases are expressed in various cellular compartments such as the lysosome, the endoplasmic reticulum, and the Golgi apparatus. The substrates of mammalian sulfatases are sulfolipids, glycosaminoglycans, and steroid hormones. These enzymes maintain neuronal function in both the central and the peripheral nervous system, chondrogenesis and cartilage in the connective tissue, detoxification from xenobiotics and pharmacological compounds in the liver, steroid hormone inactivation in the placenta, and the proper regulation of skin humidification. Human sulfatases comprise 17 genes, 10 of which are involved in congenital disorders, including lysosomal storage disorders, while the function of the remaining seven is still unclear. As for the genes responsible for pathogenesis, therapeutic strategies have been developed. Enzyme replacement therapy with recombinant enzyme agents and gene therapy with therapeutic transgenes delivered by viral vectors are administered to patients. In this review, the biochemical substrates, disease manifestation, and therapy for sulfatases are summarized.

## 1. Introduction

**Figure 1:**
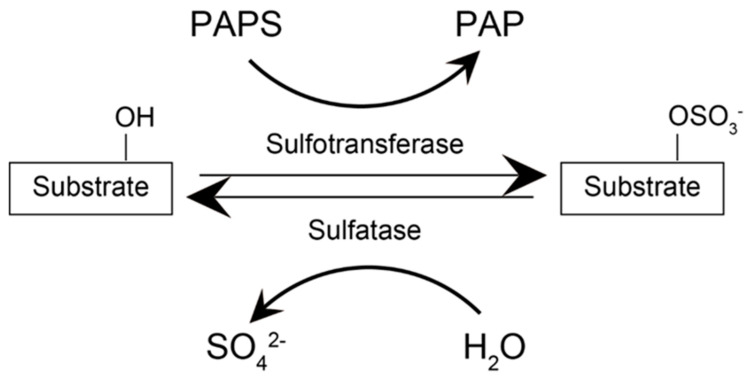
Sulfatase and sulfotransferase. Sulfotransferase transfers a sulfate at the expense of PAPS at the specific position of lipids, cholesterol, and sugars. Sulfatase removes a sulfate from the substrate. Sulfatase needs to be activated by a formylglycine-generating enzyme, as described in the text. PAPS, 3′-phosphoadenosine 5′-phosphosulfate.

Sulfate is an essential compound for the proper growth and development of living organisms [1]. Its origin is either dietary or through the biosynthesis of sulfur-containing amino acids. The latter process is known as sulfonation and involves 3′-phosphoadenosine 5′-phosphosulfate, a sulfur donor to the substrate, through the enzymatic action of sulfotransferase (Figure 1) [1]. Sulfonation modulates several biological properties of the substrate. First, the sulfonation of carbohydrates leads to glycosaminoglycans with hydrogel-like properties that are necessary for the proper maintenance of cartilage and connective tissues. In the liver, sulfonation detoxifies xenobiotics and certain pharmaceutical compounds, such as acetaminophen or paracetamol, by increasing their hydrophobicity to eliminate them in the urine. Sulfonation also inactivates several biological products induced by hormones and neurotransmitters, maintaining the homeostasis of the body. Thus, improper control of this process disturbs biological systems.

Sulfatases are enzymes that catalyze the removal of sulfate from biological substances. Currently, 17 human sulfatases have been identified (Table 1). These enzymes are activated by the formylglycine-generating enzyme (FGE), which is encoded by the sulfatase modifying factor 1 (*SUMF1*) gene. Importantly, the function of one third of the mammalian sulfatases is currently uncharacterized. Surprisingly, there are over 37,000 sulfatase genes in algae, accounting for approximately 90% of all sulfatase genes in the SulfAtlas Database [2]. Most genes in algae also require FGE as an activator. These sulfatases have two distinct domains: the large N-terminal domain with an alkaline phosphatase-like structure and three distinct domains, and the smaller C-terminal domain with a four-stranded anti-parallel β-sheet tightly packed against the N-terminal domain through an α-helix [3]. A few sulfatases present a serine rather than a cysteine at their catalytic center; these are still activated by FGE. Moreover, a limited number of sulfatases that do not require FGE for activation has been reported.

## 2. Biochemistry

Sulfatases remove sulfate from a substrate (Figure 1). Mammalian sulfatases have a broad substrate specificity (Table 2). Iduronate 2-sulfatase (IDS), arylsulfatase B (ARSB), glucosamine 6-sulfatase (GNS), heparan N-sulfatase (sulfamidase, SGSH), arylsulfatase G (ARSG), and galactosamine 6-sulfatase (GALNS) remove SO_4_^2−^ with glycosaminoglycans such as dermatan sulfate (DS), heparan sulfate (HS), and keratan sulfate. More specifically, IDS and ARSB react with DS by removing 2-O-sulfate and 6-O-sulfate, respectively. IDS, SGSH, GNS, and ARSG react with HS by removing 2-O-sulfate, N-O-sulfate, 6-O-sulfate, and 3-O-sulfate, respectively. GALNS removes 6-O-sulfate from keratan sulfate. In contrast, arylsulfatase A(ARSA) and arylsulfatase C (ARSC) react with non-carbohydrate compounds: ARSA reacts with sulfolipids, and ARSC reacts with 3-O-steroids. Currently, the substrate for arylsulfatase E (ARSE) remains uncharacterized. Although the specificity of substrates varies, the amino acid sequences of the catalytic center are well-conserved with a consensus sequence of (C/S)XPSRXXX(L/M)TG(R/K/L) [13], where C is the target cysteine residue to be converted into formylglycine. The proposed mechanisms involve the addition of an oxygen atom to the cysteine, which is activated by Cu (I) with two cysteine residues [14]. The flavin-adenine dinucleotide-containing proteins Erv2p and Ero1p have been suggested as electron donors of this redox reaction [13].

Based on biochemical data, the preferable substrate of FGE in vitro is ARSA (Table 2) [13]. Similarly, ARSB is another good substrate for FGE. The reactivity for ARSE is higher than that for ARSA. Conversely, SGSH, GALNS, and IDS are poor substrates compared to ARSA. However, it is important to remember that this assay measures the reactivity of FGE to the corresponding 23-mer peptides of sulfatases; thus, the results only warrant the biochemical reaction. To consider the physiological enzyme activity of sulfatases based on FGE-mediated activation, it is also important to consider the expression levels of FGE within specific tissues. An initial characterization of FGE revealed that this enzyme is highly expressed in the kidney and scarcely expressed in the brain [15].

In the fetal brain, nine sulfatases are abundantly expressed, with a rather broad range of substrate specificity [1]. Among them, the *IDS*, *GNS*, *ARSB*, *ARSK*, *SULF1*, and *SULF2* genes, which are responsible for proteoglycan metabolism, are highly expressed. Furthermore, the *ARSA* gene that codifies a key enzyme for the maintenance of myelination in the nerve system is also highly expressed. The *ARSC* gene, also known as the steroid sulfatase (*STS)* gene, essential for X-linked ichthyosis (XLI) of the skin and the codification of an enzyme involved in steroid metabolism by catalyzing 3-O-sulfate removal, is also highly expressed. Except for the *STS* gene, the remaining sulfatase genes are expressed in the cerebrum, brain stem, diencephalon, and cerebellum parts of the brain throughout the 4–17 weeks of the gestational period. The *ARSC* mRNA levels are low in the cerebrum when compared to the other three regions, suggesting that steroid activity is spatially confined to the latter three brain regions during early neurodevelopment. The *SULF1* and *SULF2* mRNA levels are high in the developing fetal brain. They lead to the production of an enzyme that removes 6-O-sulfate from proteoglycans extracellularly and modulates the gradient of the morphogen *Shh* during embryonic development. The same study also showed that *SUMF2* had higher expression than *SUMF1*, raising the possibility that both enzymes cooperatively regulate sulfatase activity in the brain. The short bursts of increased mRNA expression of *ARSI*, *GALNS*, and *SGSH* suggest a localized and temporary requirement for proteoglycan-metabolizing sulfatases. The abundant mRNA expression of these sulfatases implies that the metabolism of sulfur-containing substances is an active process in the developing fetal brain.

## 3. Disease Manifestations and Phenotypes in Animal Models

Mucopolysaccharidoses (MPSs) are a group of congenital disorders presenting an accumulation of mucopolysaccharides, such as DS, HS, keratan sulfate, and chondroitin sulfate [16]. Visceral manifestations are widely prevalent in MPSs. Additionally, HS accumulation caused by a deficiency of IDS (MPS II), SGSH (MPS IIIA), and GNS (MPS IIID) is related to neurological manifestations, such as cognitive decline. In contrast, DS accumulation is associated with a deficiency of IDS (MPS II) and ARSB (MPS VI), leading to skeletal manifestations such as short stature, increased head circumference, claw hand, and coarse facial features. MPS IVA is caused by a deficiency of GALNS that affects the bones and the heart. Interestingly, the affected individuals show normal growth at birth, presenting these developmental defects only at age 4–5.

Metachromatic leukodystrophy (MLD) is caused by a failure of myelination homeostasis by a deficiency of the ARSA enzyme in the central (CNS) and the peripheral (PNS) nervous system, leading to demyelination and, consequently, devastating neural manifestations. The disease subtypes are defined by the age of onset and are categorized into infantile-, juvenile-, and adult-onset. Patients with infantile-onset MLD, the most severe subtype, show symptoms within the first two years of life and decease within a few years [17].

Chondrodysplasia punctate 1 is caused by a pathogenic mutation in the *ARSE* gene that causes short stature and the calcification of the epiphyseal cartilage and its neighboring area [18]. XLI, also known as steroid sulfatase deficiency, is caused by a pathogenic mutation in the *ARSC* gene, leading to skin disorders [19,20]. A deficiency of the *ARSC* gene results in an abnormal accumulation of cholesterol 3-O-sulfate, leading to a loss of proper moisture of the skin. A deficiency of the STS enzyme in the placenta causes difficulties in labor due to a limited accumulation of the hormone dehydroepiandrosterone sulfate.

Lysosomal storage disorders (LSDs) are congenital disorders characterized by an accumulation of specific metabolites that are degraded by lysosomes [21]. Approximately 50 genes are implicated in various LSDs, including MPSs, sphingolipidoses, neuronal lipofuscinosis, and glycogen storage disorders; additionally, other genes are related to lysosomal biogenesis, membrane proteins with transporter function, and enzymes responsible for the maturation of LSD-involved enzymes. Major phenotypes of LSDs include neuronal, skeletal, and visceral manifestations that emerge at earlier ages. Among the aforementioned disorders, MPS and MLD are included in LSDs.

The phenotypes for Sulf1/Sulf2 have been also described in animal models. These enzymes locate extracellularly to remove a sulfate at the 6-O position of GlcNAc at a neutral pH. A deficiency of these enzymes leads to embryonic development and the chondrogenic phenotype. In Sulf1(-/-) mice, the differentiation of neural progenitors to neurons is enhanced.

## 4. Treatment Strategies

In the context of treatment, pathogenic cells need to be restored to the original non-pathogenic conditions. To achieve this, a variety of strategies have been developed. Enzyme replacement therapy provides therapeutic enzymes to the cells, while gene therapy transduces cDNA to the pathogenic cells. Hematopoietic stem cell transplantation exerts its therapeutic effects by transplanting hematopoietic stem cells that generate mature cells, such as lymphocytes, erythrocytes, platelets, and other myeloid cells. Additionally, pharmacological chaperons that are low-molecular-weight compounds play a substantial role in correcting dysfunctional pathogenic enzymes.

Cross-correction is an important mechanism for the regulation of enzymes related to LSDs. LSD-related enzymes target the lysosome via the mannose-6-phosphate-mediated intracellular traffic mechanism, while exogenously administered enzymes also reach lysosomes by this mechanism. This implies that bone marrow transplants, enzyme replacement therapy, and gene therapy do not necessarily transduce therapeutic cDNA into the pathogenic cells. Alternatively, once these active enzymes are expressed in the cells adjacent to the pathogenic cells, the treatment effect is exerted by cross-correction.

### 4.1. Enzyme Replacement Therapy

Enzyme replacement therapy was initially developed for Gaucher disease and was then applied to other LSDs. Although, in the early phase, therapeutic enzymes were purified from the placenta, currently the available commercial products are produced under controlled conditions and with an authorized procedure. Therapeutic enzymes are usually administered fortnightly to treat visceral symptoms. Currently, the treatment of CNS manifestations is the focus of research. To achieve this, a variety of delivery protocols other than intravenous administration, e.g., intrathecal administration [22], have been developed. Moreover, a method to efficiently transduce enzyme agents to the brain has been developed with an enzyme agent fused to the Fab domain of the anti-human transferrin receptor of the enzyme’s N-terminal [23]. Another enzyme agent that links to the Fc domain of the monoclonal anti-human transferrin receptor antibody through the enzyme’s C-terminal has been also developed [24].

### 4.2. Gene Therapy

Gene therapy is an emerging technique through which a transgene is delivered into the body by viral-vector-mediated technology. Compared to enzyme replacement therapy, which requires regular administration, gene therapy is considered a one-time therapy that relieves patients from frequent medical visits.

#### 4.2.1. Adeno-Associated Virus (AAV)

AAV is a widely used vector for gene therapy [25]. AAV was originally discovered as an associated virus of adenoviruses more than five decades ago. AAV is a family of parvoviruses that infect humans with unknown pathogenicity. Therapeutic AAV carries transgenes of up to approximately 5.5 kb. There are many serotypes with different tropisms. For example, AAV9 infects neural cells, whereas AAV2 infects the liver. Additionally, in many hybrid AAVs, the serotypes of the capsid and the cloning vector are different. The tropism depends on the amino acid sequence of capsid proteins. It has been demonstrated that the replacement of tyrosine with phenylalanine dramatically increases the efficiency of AAV transduction [26]. Usually, viral particles are prepared by transfecting three plasmids that express cDNA (a cloning vector), capsid proteins, and a replicase enzyme. For further purification, the produced AAV particle is isolated using ultracentrifugation and/or ion exchange chromatography. In addition to no severe adverse events, the biosafety of AAV has also been extensively studied in many non-human primate models [27].

For improved therapeutic outcomes, the administration of a larger amount of AAV particles is needed; however, this occasionally leads to hepatic toxicity. To reduce the amount of the vector, Stristava et al. [26] replaced threonine with phenylalanine in the capsid protein VP-3, leading to enhanced AAV stability. Regarding the production of gene-delivery medicines at a large scale, ultracentrifugation using cesium chloride limits the capacity of sample processing. Hence, this technique has been replaced by the iodixanol-mediated protocol [25]. Based on these technical improvements, AAV has been established as a therapeutic agent for gene therapy.

#### 4.2.2. Lentiviral Vector (LV)

LV is another viral vector widely used in therapeutics and for ex vivo gene transduction [28]. LV originated from the HIV-1 virus and was then developed into a non-self-replicating viral vector. Currently, the available plasmid system uses three plasmids, namely, the transgene, helper, and capsid plasmids. Third-generation vectors lack accessory genes and part of the native U3 promoter. Apart from these advances, gene therapy researchers investigate more adequate promoters, modifiers, and the 3′-UTR region that may enhance the stability of the transcript [29]. Despite the possibility of multiple applications, LV-mediated gene therapy is mostly used for autologous transplantation. Thus, if the medical facility where the procedure takes place is not easily accessible to the patient, this may be problematic. Currently, research on the efficient transportation of gene-transfected cells is under examination. Apart from LSDs, the use of LV in immunology and oncology has been growing. For instance, chimeric antigen receptor-T therapy delivers a modified receptor specific to antigens against pathogenic T cells.

## 5. Gene Therapy for Sulfatases

Among the 17 human sulfatases, *ARSA*, *ARSB*, *GALNS*, *GNS*, *SGSH*, and *IDS* are involved in LSDs for which gene therapy studies have been performed (Table 1). Here, we present a summary of the current status of relevant preclinical and clinical research. Some unique issues, such as the phenotype of the disorder and the choice of AAV serotype, animal models, and vector organization, are also described.

### 5.1. ARSA

ARSA is an enzyme responsible for MLD (OMIM 250100). MLD is a detrimental disorder that affects both the CNS and PNS. Compared to other LSDs, MLD is the best-studied sulfatase deficiency for gene therapy (Table 3). A pilot study of newborn screenings for MLD has been reported [30]. In this context, the substrate for the measurement of ARSA’s enzymatic activity in dried blood spots by liquid chromatography–tandem mass spectrometry has been developed [31].

Ex vivo gene therapy for MLD is a success in this field (reviewed in [28]). Although *Arsa*-deficient mice exhibit milder phenotypes than humans, impaired neurophysiology, rotarod latency, and behavioral data were documented at 6 months of age compared to age-matched controls [4,31]. LV-mediated hematopoietic stem-cell gene therapy in *Arsa*-deficient mice showed a supraphysiological ARSA enzyme activity in the liver and peripheral blood and a marginal elevation in the brain [4]. In detailed histological analyses, the reduced number of neurons in *Arsa*-deficient mice in the CA2/3 region of the hippocampus and the Purkinje cell layer of the cerebellum was reversed by LV-mediated gene therapy. The results of subsequent clinical studies revealed that the MRI evaluation was improved [17,32,33]. In the first report of a relevant clinical study [31], (i) the vectors were manufactured with authorized protocols, (ii) the vector copy number reached acceptable levels for treatment, (iii) an improved MRI evaluation of the brain was observed, and (iv) the integration sites of the vector in the chromosomes identified in three patients were similar to LV-treated patients with X-linked adrenoleukodystrophy with a partially overlapping pattern. Consequently, the LV-based therapeutic agent Libmeldy was approved by the European Medical Agency and is now commercially available by Orchard Therapeutics, London, UK.

Gene therapy for MLD started with AAV-mediated transduction (Table 3). Numerous animal studies established that an AAV-mediated enhancement of ARSA enzyme activity is beneficial. Importantly, the discussion related to the delivery or the site of infusion is ongoing. An initial attempt suggested the intravenous injection of AAV. In the case of MLD, various injection sites in the brain have been examined. Because the administered AAV stays at or near the site of injection and fails to easily diffuse, an altered injection protocol for the brain should be developed.

ARSA is a lysosomal sulfatase that is activated by multiple steps in the cells. After translation in the endoplasmic reticulum, the immature protein first passes through the Golgi apparatus, where the cysteine at the catalytic center is converted to formylglycine by the enzymatic action of FGE [34]. Failure in this process leads to multiple sulfatase deficiency, a known LSD with an elevation of all LSD-related sulfatases. In the context of gene therapy, the maximization of enzyme activity needs to be pursued. For this, the role of the *SUMF1* gene has been investigated in vitro and in vivo. The simultaneous expression of both *ARSA* and *SUMF1* bicistronically [35] or a vector system containing a mixture of *ARSA* and *SUMF1* [36] were examined. In both cases, enhanced *ARSA* gene expression was observed.

### 5.2. ARSB

ARSB is an enzyme responsible for MPS type VI (MPS VI: OMIM 253200), with elevated DS levels in the body being a hallmark of the disease. The major manifestation involves skeletal deformity, while no CNS effects have been observed. Even though the disease is global, many patients are located in northeastern Brazil [90]. The hybrid vector AAV2/8 was used in a preclinical study to target the skeletal tissue of the body. To examine the proper site of injection and the amount of vector, the efficacy of therapy, the safety profile of the vector, and other preclinical parameters, many animal models were used, including rats, cats, and mice. Animal models other than mice were also used to study other sulfatases of LSDs [91] because of their size.

### 5.3. GALNS

GALNS is an enzyme responsible for MPS type IVA (MPS IVA: OMIM 253000), also known as Morquio A syndrome. Its major symptoms are manifested in the bones and the visceral organs but not in the CNS. In an animal study, the tested AAV8-based vector provided prolonged expression of GALNS enzyme activity for over 6 months after administration, ameliorating the accumulation of keratin sulfate and improving a variety of pathological markers in the growth plate and the articular disc of the knee joint as well as the valve and muscle of the heart [59].

### 5.4. SGSH

SGSH is an enzyme responsible for MPS type IIIA (MPS IIIA: OMIM 252900). Among the four disease subtypes of MPS III, this is the most frequently observed. MPS IIIA patients are principally found in Europe, particularly the Netherlands, but there are many patients globally [92]. For MPS IIIA, both AAV and LV gene therapies have been developed. As MPS IIIA exhibits a severe CNS phenotype but not skeletal symptoms, the delivery protocol of the transgene into the brain has been extensively studied. In the majority of animal studies, the site of AAV injection was examined to optimize the therapeutic outcomes (Table 3). In contrast, ex vivo transduction was selected for the LV treatment.

Similar to ARSA, the role of FGE has also been investigated in *SGSH* gene transduction [35]. First, the expression of *SUMF1* led to an enhanced SGSH enzymatic activity in several in vitro experiments. This was also true for in vivo experiments where the SGSH enzyme activity was measured in the brain. In this experiment, the SGSH enzymatic activity in the brain was studied with or without the SUMF1 enzyme under the PGK promoter or the CAG promoter. Under the PGK promoter, the co-expression of this modifier enhanced SGSH enzymatic activity, while under the CAG promoter, the effect on the SGSH enzyme activity was drastic. Based on these data, a therapeutic vector with a CAG promoter for the *SGSH* transgene is currently being developed (NCT03612869).

To enhance the expression of the SGSH enzyme, the effect of the signal peptide IDS (1-33) was studied since it induces a strong secretion of proteins into the extracellular space [62]. In this case, the anticipated results were obtained.

### 5.5. IDS

IDS is an enzyme responsible for MPS II (OMIM 309900). Its major phenotype involves CNS, skeletal deformity, and visceral manifestations. About 70% of the patients have the severe type of MPS II, presenting CNS-related symptoms [93] and a mutation of recombination or large deletion of the *IDS* gene because of the pseudogene *IDS2* located on the telomeric side of chromosome X. The remaining 30% suffer from the attenuated type of MPS II, which does not affect the CNS. Both AAV and LV gene therapies have been investigated in MPS II murine models. Similar to MLD, in MPS IIIA, the visceral manifestations are controlled under the existing intervention protocols. Although CNS manifestations are challenging, recent data showed that enhanced IDS enzymatic activity improves the brain-specific symptoms [80,82]. Similarly, a beneficial effect of LV therapy for bone manifestations has been documented [88], indicating that increased expression of the transducing gene contributes to a better therapeutic outcome.

An AAV gene therapy product is in clinical trials by REGENEXBIO Inc., (Rockville, MD, USA). This is to be injected into the brain, aiming to improve the pathology (NCT04571970). The reported animal study showed promising results; thus, similar outcomes might be obtained with the clinical study.

Moreover, Sangamo Therapeutics, Inc. provides a unique product using zinc finger technology [94]. Briefly, they use three AAV vectors for gene editing. The first two vectors express two zinc fingers targeting the albumin locus of the hepatocyte. The third vector expresses the donor sequence of the transgene. Under optimal conditions where all three vectors are expressed in a single cell in the liver, expression of the *IDS* gene under the endogenous albumin promoter is expected [83]. Currently, a clinical trial is ongoing (NCT03041324).

## 6. Emerging Techniques

### 6.1. Lipid Nanoparticle (LNP)

LNP is a non-viral delivery method for mRNA-based medicine [95]. The history of LNP research originates in the study of liposomes, but this innovative technology that is directly applicable to gene therapy has advanced in recent years. Still, there are several areas for improvement. First, the lipid used for LNP is carefully examined. Based on synthetic chemistry, synthetic lipids, collectively called cation-ionizable lipids, show great potential for gene delivery in vivo because of their long life, low toxicity, and the potential for the modification of the LNP surface by antibodies, leading to the production of a sophisticated drug delivery system of transgenes with specific tropisms [96].

The development of this technology began before the mRNA vaccine against COVID-19, the pandemic that emerged in late 2019. Another reason for the ease of in vivo use of this technology is the advances in stabilization technology for mRNA. Generally, mRNA is thought to be more fragile than DNA due to the presence of RNases in the laboratory setting. However, recent advances in the improvement of mRNA stability solved several of these issues. One issue of mRNA instability relates to its 5’-cap structure. A new technology that replaces the wild-type ribonucleic acid with a 2’-O-methoxy analog enhances mRNA stability. Moreover, this replacement makes the product resistant to degradation by RNases. For example, pseudouridine is a uridine analog found at a proportion of approximately 5% compared to uridine. Due to the impaired susceptibility of pseudouridine-containing mRNA to RNase, this modified mRNA has been used for the synthesis of RNA with a prolonged half-life in vivo. Additionally, the length of poly-A affects the cellular half-life of mRNA. The optimization of these multiple parameters in silico can convert the prototypical mRNA delivery agent into a valuable final product.

### 6.2. Gene Delivery

For safety reasons, intravenous administration has often been chosen for gene delivery. In LSD treatment, the brain is a key target. In this case, the direct injection of agents into the brain is needed. To administer a relatively large amount of agents across an entire brain area, intrathecal, intracisternal, and intracerebroventricular administration have been used [97] (Table 3). Apart from these delivery techniques, the intranasal administration of drugs has also been investigated. After intranasal administration, the enzymatic activity in the olfactory bulb recovered to 100-fold compared to the control, maintaining the same levels in the rest of the brain [98]. Recent studies reported the efficacy of the in utero administration of viral vectors in the mouse, demonstrating promising outcomes [99,100].

### 6.3. Peptide Modifiers

In the last decades, efforts to screen peptides that enhance the penetration of drugs through the blood–brain barrier have been made, reporting numerous such peptides [101]. The relevant sequences of these peptides are found in the part of the endogenous protein that penetrates the blood–brain barrier either by itself or by a receptor–ligand mechanism. Zhang et al. [102] recently reported that the dodecapeptide PB5-3 enhances the transduction of AAV9 to the brain. The sequence of this peptide has no similarity to known sequences of proteins/peptides, with approximately 2- to 3-fold increases in AAV9 transduction in the brain compared to intravenous administration. The detailed mechanism of this effect remains to be elucidated. Furthermore, the combination of the AAV serotype and the peptide sequence plays an essential role in determining the efficacy of the peptide modifiers.

### 6.4. CRISPR/Cas9

The genetic correction of pathogenic genes is becoming an essential technology in research and medicine. A recent study reported a CRISPR/Cas9 correction of the *ARSA* gene in vitro [103]. In this experiment, patient-derived hematopoietic stem/progenitor cells were treated with five guide RNAs (gRNAs) at the 5′-UTR or in close proximity to the transcription start site of the *ARSA* gene, which allowed the recombination of codon-optimized therapeutic *ARSA* cDNA using AAV6. The optimization of the experimental setting included (i) gRNA sequences, (ii) multiplicity of infection, and (iii) the molar ratio of the ribonuclease protein consisting of Cas9 protein and gRNA. The authors reported that 16.6–37% of the GFP^+^ transduced cells were detected by FACS analysis, and a 19- to 32-fold increase in ARSA activity was documented with an enzyme assay. The advantages of this technique include the following: (i) a single set of pre-examined gRNAs that cleaves at the 5′-UTR or in close proximity to the transcription start site of the gene of interest can be used for many pathogenic genes; (ii) due to the insertion at a known locus by CRISPR/Cas9, there is no possibility of random integration when LV is used; (iii) the use of a codon-optimized therapeutic cDNA allows for maximal enzyme expression; and (iv) the use of an exogenous gene (e.g., the SV40 poly-A region) in the repair template prevents a second cleavage after the recombination. A similar technique has been used in immunodeficiency (SCID-X1), Wiskott–Aldrich syndrome, and severe congenital neutropenia [104,105,106]. However, this novel protocol for the *ARSA* gene uses lower amounts of the vector at only two orders of magnitude.

## 7. Future Perspectives

A growing number of gene therapy products have recently been developed. Both AAV and LV are established vehicles of gene therapy. A known disadvantage of AAV is the amount of the therapeutic vector required for effective treatment. Innovations to reduce this burden, such as the threonine-to-phenylalanine replacement described by Zhong et al. [26], may help improve this situation. Moreover, the production of antibodies against the capsid protein of AAV is confounding. For LV, the application is largely limited to hematopoietic stem cell transplantation. However, these are relatively minor issues compared to the fact that no known severe adverse events have been reported for these vectors. Further research on emerging techniques may help improve the vectors’ safety and application.

## Figures and Tables

**Table 1:** Sulfatases in humans and mice.

Sulfatase	Human Gene	OMIM	Genetic Disorder	Mouse Gene	Phenotype	Ref.
Arylsulfatase A	*ARSA*	250100	Metachromatic leukodystrophy (MLD)	*Arsa*	CNS and PNS involvement	[4]
Arylsulfatase B	*ARSB*	253200	Maroteaux–Lamy syndrome (MPS VI)	*Arsb*	Bone deformity, visceral manifestation	[5]
Arylsulfatase C (Steroid sulfatase)	*ARSC*, *STS*	308100	X-linked ichthyosis (XLI); difficult labor	*Arsc*	Not reported	
Arylsulfatase D	*ARSD*	300002	Not reported	Not identified	Not applicable	
Arylsulfatase E	*ARSE*	302950	Chondrodysplasia punctata 1 (CDPX1)	*Arse*	Not reported	
Arylsulfatase F	*ARSF*	300003	Not reported	Not identified	Not applicable	
Arylsulfatase G	*ARSG*	618144	Usher syndrome type IV	*Arsg*	Neuronal cell death and behavioral deficits	[6]
Arylsulfatase H	*ARSH*	300586	Not reported	Not identified	Not applicable	
Arylsulfatase I	*ARSI*	610009	Not reported	*Arsi*	Not reported	
Arylsulfatase J	*ARSJ*	610010	Not reported	*Arsj*	Not reported	
Arylsulfatase K	*ARSK*	610011	Mucopolysaccharidosis type 10	*Arsk*	Mild behavioral changes	[7]
Galactosamine 6-sulfatase	*GALNS*	253000	Morquio A syndrome (MPS IVA)	*Galns*	Bone deformity, visceral manifestation	[8]
Glucosamine 6-sulfatase	*GNS*	252940	Sanfilippo D syndrome (MPS IIID)	*Gns*	CNS phenotype, visceral manifestation	[9]
Heparan N-sulfatase (sulfamidase)	*SGSH*	252900	Sanfilippo A syndrome (MPS IIIA)	*Sgsh*	CNS phenotype, visceral manifestation	[10]
Iduronate 2-sulfatase	*IDS*	309900	Hunter syndrome (MPS II)	*Ids*	CNS phenotype, bone deformity, visceral manifestation	[11]
Sulfatase 1	*SULF1*	610012	Not reported	*Sulf1*	Short life span, skeletal and renal abnormalities	[12]
Sulfatase 2	*SULF2*	610013	Not reported	*Sulf2*	Short life span; skeletal and renal abnormalities	[12]

OMIM, Online Mendelian Inheritance in Man.

**Table 2:** Subcellular localization and substrate of sulfatases.

Sulfatase	Subcellular Localization	Substrate
*ARSA*	Lysosome	Sulfatide
*ARSB*	Lysosome	4-O; DS
*ARSC*	ER/microsome	DHEAS, estriol sulfate, pregnolone sulfate, cholesterol sulfate
*ARSD*	ER	Uncharacterized
*ARSE*	Golgi	Uncharacterized
*ARSF*	ER	Uncharacterized
*ARSG*	Lysosome	3-O; HS
*ARSH*	Uncharacterized	Uncharacterized
*ARSI*	Uncharacterized	Uncharacterized
*ARSJ*	Uncharacterized	Uncharacterized
*ARSK*	Uncharacterized	2-O; HS, CS
*GALNS*	Lysosome	6-O; KS, CS
*GNS*	Lysosome	6-O; HS
*SGSH*	Lysosome	N-O; HS
*IDS*	Lysosome	2-O; DS/HS
*SULF1*	Extracellular	6-O; HS
*SULF2*	Extracellular	6-O; HS

DHEAS, dehydroepiandrosterone sulfate.

**Table 3:** Vectors used for the study of sulfatase gene therapy.

Sulfatase	Disorder	Year	Vector	Promoter	Transgene	3′-UTR	Animal	Dose	Route	Ref
ARSA	MLD	2006	AAV5	PGK	*ARSA*	WPRE	Mouse	3 × 10^9^ particles	Brain	[37]
		2009	AAV1	CAG	*ARSA*		Mouse	5 × 10^10^ particles	Intrathecal	[38]
		2012	AAVrh	CAG	*ARSA*		Mouse	2.3 × 10^9^ vg	Intravenous	[39]
		2012	AAV5	PGK	*ARSA*		Mouse	2.3 × 10^9^ vg	Intravenous	[39]
		2014	AAVrh10	CMV	*ARSA*		NHP	1.5 × 1012 gc	Brain	[40]
		2014	ssAAV9	Not described	*ARSA*		Mouse	2 × 10^12 ^ vg	Intravenous	[41]
		2015	AAV1	CAG	*ARSA*		Mouse	2.3 × 10^11^ vg	Brain	[42]
		2015	AAV9	CAG	*ARSA*		Mouse	1.1 × 10^10^ vg	Brain	[42]
		2015	scAAV1	CAG	*ARSA*		Mouse	1.1 × 10^10^ vg	Brain	[42]
		2021	AAVPHP.eB	CAG	*ARSA*		Mouse	5 × 10^11^ vg	Intravenous	[43]
		2021	AAV9	CAG	*ARSA*		Mouse	4.0 × 10^11^ vg	Intrathecal	[44]
		2015	AAVrh10	Not described	*ARSA*		NHPs	1.1 × 10^11^ vg total	Brain	[45]
		2021	AAVrh10	Not described	*ARSA*		NHPs	0.0285 or 1.5 × 10^12^ gc	Brain	[27]
		2001	LV	CMV	*ARSA*	WPRE	Mouse	80–200 ng p24 equivalent	Brain	[46]
		2005	LV	CMV	*ARSA*	WPRE	Mouse	80 ng p24 equivalent	Brain	[47]
		2006	LV	hPGK	*ARSA*	WPRE	Mouse	MOI = 100; 10^6^ cells	Intravenous	[4]
		2007	LV	PGK	*ARSA*	WPRE	Human HSPC	MOI = 100	Intravenous	[48]
		2010	LV	PGK	*ARSA*	WPRE	Mouse	2 × 10^6^ total unit	Brain	[49]
		2014	LV	EF1	*ARSA*	WPRE	Mouse	2.5 × 10^7^ total unit	Brain	[50]
		2013	LV	hPGK	*ARSA*	WPREmut6	Human	MOI = 100; 2–10 × 10^6^/mL	Intravenous	[17]
ARSB	MPS VI	2014	AAV2/8	TBG	*ARSB*		Mouse	2 × 10^12^ gc/kg	Intravenous	[51]
		2016	AAV2/8	TBG	*ARSB*		Mouse	2–6 × 10^11^ gc/kg	Intravenous	[52]
		2017	AAV2/8	TBG	*ARSB*		Mouse	2 × 10^11^–2 × 10^12^ gc/kg	Intravenous	[53]
		2020	AAV2/8	TBG	*ARSB*		Mouse	0.2 or 2 × 10^13^ gc/kg	Intravenous	[54]
		2008	AAV2/8	TBG	*ARSB*		Rat	4.1 × 10^13^ gc/kg	Intravenous	[55]
		2008	AAV2/1	CMV	*ARSB*		Rat	3.6 × 10^13^ gc/kg	Intramuscular	[55]
		2002	AAV2	CAG	*ARSB*		Cat	0.56–1.1 × 10^9^ particles	Intraocular	[56]
		2008	AAV2/8	TBG	*ARSB*		Cat	6.6 × 10^13^ gc/kg	Intravenous	[55]
		2008	AAV2/1	CMV	*ARSB*		Cat	4.3 × 10^12^ gc/kg	Intramuscular	[55]
		2011	AAV2/8	TBG	*ARSB*		Cat	0.2–6 × 10^13^ gc/kg	Intravenous	[57]
		2013	AAV2/8	TBG	*ARSB*		Cat	0.22 × 10^12^ gc/kg	Intravenous	[58]
		2020	AAV2/8	TBG	*ARSB*		Cat	2 × 10^12^ gc/kg	Intravenous	[54]
GALNS	MPS IVA	2020	AAV8	TBG	*GALNS*	RBG	Mouse	5 × 10^13^ gc/kg	Intravenous	[59]
		2020	AAV8	TBG	*D8-GALNS*	RBG	Mouse	5 × 10^13^ gc/kg	Intravenous	[59]
		2021	AAV9	CAG	*GALNS*		Rat	6.67 × 10^13^ vg/kg	Intravenous	[60]
GNS	MPS IIID	2017	AAV9	CAG	*GNS*		Mouse	5 × 10^10^ vg	Cisterna magna	[9]
SGSH	MPS IIIA	2007	AAV2/5	CMV	*SGSH-IRES-SUMF1*		Mouse	0.6–3 × 10^10^ particles	Brain	[61]
		2019	AAV9	CMV	*IDS(1-33)-SGSH-* *SUMF1*		Mouse	5.4 × 10^12^ gc/kg	Cisterna magna	[62]
		2015	scAAVrh74	U1a	*hSGSH*		Mouse	5 × 10^12^ gc/kg	Intravenous	[63]
		2016	scAAV9	U1a	*hSGSH*		Mouse	1–5 × 10^12^ gc/kg	Intravenous	[64]
		2016	AAVrh10	PGK	*SGSH-IRES-SUMF1*		Mouse	7.5 × 10^9^ gc	Brain	[10]
		2018	AAV4	CMV	*SGSH*		Mouse	5 × 10^10^ particles	Lateral ventricles	[65]
		2018	AAV4	CMV	*SGSHv4*		Mouse	5 × 10^10^ particles	Lateral ventricles	[65]
		2019	AAVrh10	CAG	*SGSH*		Mouse	0.086–9.0 × 10^10^ vg	Brain	[66]
		2019	AAVrh10	PGK	*SGSH-IRES-SUMF1*		Mouse	4.1 × 10^9^ particles	Brain	[35]
		2019	AAVrh10	PGK	*SGSH*		Mouse	4.1 × 10^9^ particles	Brain	[35]
		2019	AAVrh10	CAG	*SGSH*		Mouse	4.1 × 10^9^ particles	Brain	[35]
		2020	scAAV9	mCMV	*SGSH*	SV40 polyA	Mouse	0.25–5 × 10^13^ vg/kg	Intravenous	[67]
		2021	scAAV9	U1A	*SGSH*		Mouse	3 × 10^13^ vg/kg	Intravenous	[68]
		2019	AAVrh10	CAG	*SGSH*		NHP	7.2 × 10^11^ vg	Brain	[66]
		2019	AAVrh10	CAG	*SGSH*		Dog	1–2 × 10^12^ vg	Brain	[66]
		2019	AAV9	CMV	*IDS(1-33)-SGSH-SUMF1*		Pig	4.5 × 10^12^ gc/kg	Cisterna magna	[62]
		2014	AAVrh.10	PGK	*SGSH-IRES-SUMF1*		Human	7.2 × 10^11^ vg	Brain	[69]
		2012	LV	SFFV	*SGSH*	WPRE	Mouse	1.5–2.5 × 10^5^ Lin^−^ cells	Intravenous	[70]
		2013	LV	hCD11b	*SGSH*	WPRE	Mouse	MOI = 30; 0.2–1 × 10^5^ cells	Intravenous	[71]
		2013	LV	hPGK	*SGSH*	WPRE	Mouse	MOI = 30; 0.2–1 × 10^5^ cells	Intravenous	[71]
		2014	LV	EF1a	*SGSH*		Mouse	Not described	Brain	[72]
		2014	LV	EF1a	*SGSH-SUMF1*		Mouse	Not described	Brain	[72]
		2019	LV	CD11b	*SGSH*		Mouse	MOI = 60; 3 × 10^5^ Lin^−^ cells	Intravenous	[73]
		2010	canine Ad serotype 2	CMV	*SGSH-IRES-GFP*	PolyA	Mouse	6 × 10^9^ particles	Brain	[74]
		2012	canine Ad	RSV	*SGSH-IRES-GFP*		Mouse	2 × 10^9^ particles	Brain	[75]
IDS	MPS II	2006	AAV2/8	TBG	*IDS*		Mouse	1 × 10^11^ particles	Intravenous	[76]
		2009	AAV2/5	CMV	*IDS*		Mouse	1 × 10^11^ particles	Intravenous	[77]
		2010	AAV2/8	EF	*IDS*	WPRE	Mouse	1 × 10^11^ particles	Intravenous	[78]
		2016	AAV9	CB	*IDS*	RBG	Mouse	3 × 10^8^–3 × 10^10^ gc	Intracerebroventricular	[79]
		2016	AAV9	CAG	*IDS*		Mouse	5 × 10^10^ vg	Intracisternal	[80]
		2017	AAV9	CB7	*IDS*	RBG	Mouse	5.6 × 10^10^ vc	Intrathecal or Intracerebroventricular	[81]
		2017	AAV9	CB7	*IDS*	RBG	Mouse	5.6 × 10^10^ vc	Intravenous	[81]
		2017	AAV9	CB7	*IDS-SUMF1*	RBG	Mouse	5.6 × 10^10^ vc	Intrathecal	[81]
		2017	AAV9	CB7	*IDS-SUMF1*	RBG	Mouse	5.6 × 10^10^ vc	Intravenous	[81]
		2017	AAV9	CB7	*IDS-SUMF1*	RBG	Mouse		Intracerebroventricular	[81]
		2018	scAAV9	Mini-CMV	*IDS*		Mouse	0.25–2 × 10^13^ vg/kg	Intravenous	[82]
		2018	AAV2/8	ApoE-hAAT	*ZFNs + hIDS donor*	PolyA	Mouse	0.25–1.5 × 10^12^ vg	Intravenous	[83]
		2018	AAV9	Not described	*IDS*		NHP	1.7–5.0 × 10^13^ gc	Cisterna Magna	[84]
		2018	AAV9	Not described	*IDS*		NHP	1.7–5.0 × 10^13^ gc	Suboccipital puncture	[84]
		2015	LV	MCU3	*IDS*		Mouse	MOI = 50; 2 × 10^6^ Lin^−^ cells	Intravenous	[85]
		2018	LV	hCD11b	*IDS*	WPRE	Mouse	MOI = 100; 3–4 × 10^5^ HSCs	Intravenous	[86]
		2018	LV	hCD11b	*IDS-ApoEII*	WPRE	Mouse	MOI = 100; 3–4 × 10^5^ HSCs	Intravenous	[86]
		2020	LV	MCU3	*IDS*		Mouse	1.25 × 10^6^ cells	Intravenous	[87]
		2020	LV	MCU3	*IDS*		Mouse	6.6–7.5 × 10^5^ cells	Intravenous	[88]
		2019	LNP	Not applicable	*IDS*		Mouse	1.5 × 10^12^ vg	Intravenous	[89]

AAT, human alpha 1-antitrypsin; Ad, adenovirus; BGH, bovine growth hormone polyA; CB, chicken β-actin promoter plus CMV enhancer; gc, genome copy; NHP, non-human primate; RGB, rabbit β-globin, polyA; vg, vector genome.
